# Obesity Increases Mitogen-Activated Protein Kinase Phosphatase-3 Levels in the Hypothalamus of Mice

**DOI:** 10.3389/fncel.2017.00313

**Published:** 2017-10-09

**Authors:** Bárbara de A. Rodrigues, Vitor R. Muñoz, Gabriel K. Kuga, Rafael C. Gaspar, Susana C. B. R. Nakandakari, Barbara M. Crisol, José D. Botezelli, Luciana S. S. Pauli, Adelino S. R. da Silva, Leandro P. de Moura, Dennys E. Cintra, Eduardo R. Ropelle, José R. Pauli

**Affiliations:** ^1^Laboratory of Molecular Biology of Exercise, University of Campinas (UNICAMP), São Paulo, Brazil; ^2^Post-Graduate Program in Movement Sciences, São Paulo State University (Unesp), Institute of Biosciences, São Paulo, Brazil; ^3^OCRC—Obesity and Comorbidities Research Center, University of Campinas (UNICAMP), São Paulo, Brazil; ^4^School of Physical Education and Sport of Ribeirão Preto, University of São Paulo (USP), São Paulo, Brazil; ^5^CEPECE—Center of Research in Sport Sciences, School of Applied Sciences, University of Campinas (UNICAMP), São Paulo, Brazil; ^6^Laboratory of Nutritional Genomics, University of Campinas (UNICAMP), São Paulo, Brazil

**Keywords:** MKP-3, insulin, hypothalamus, obesity, food intake

## Abstract

Mitogen-activated Protein Kinase Phosphatase 3 (MKP-3) has been involved in the negative regulation of insulin signaling. The absence of MKP-3 is also associated with reduced adiposity, increased energy expenditure and improved insulin sensitivity. The MKP-3 is known as the main Erk1/2 phosphatase and FoxO1 activator, which has repercussions on the gluconeogenesis pathway and hyperglycemia in obese mice. Recently, we showed that MKP-3 overexpression decreases FoxO1 phosphorylation in the hypothalamus of lean mice. However, the hypothalamic interaction between MKP-3 and FoxO1 during obesity was not investigated yet. Here, the MKP-3 expression and the effects on food intake and energy expenditure, were investigated in high-fat diet-induced obese mice. The results indicate that obesity in mice increased the MKP-3 protein content in the hypothalamus. This hypothalamic upregulation led to an increase of food intake, adiposity, and body weight. Furthermore, the obese mice with increased MKP-3 showed an insulin signaling impairment with reduction of insulin-induced FoxO1 and Erk1/2 phosphorylation in the hypothalamus. Moreover, a bioinformatics analysis of data demonstrated that hypothalamic MKP-3 mRNA levels were positively correlated with body weight and negatively correlated to oxygen consumption (VO_2_) in BXD mice. Taken together, our study reports that obesity is associated with increased protein levels of hypothalamic MKP-3, which is related to the reduction of FoxO1 and Erk1/2 phosphorylation in the hypothalamus as well as to an increase in body weight and a reduction in energy expenditure.

## Introduction

The obesity is one of the most severe problems of public health worldwide nowadays. The hypothalamic tissue has been a target of an intense investigation by the scientific community in the last years as an important center regulator of energy homeostasis and hunger control, which is mediated in part by insulin (Arruda et al., 2011; Tran et al., 2016). Disruptions in insulin signal transduction in the hypothalamus caused by low-grade inflammation associated with the increase of adipose tissue constitute an important trigger for the obesity genesis (Arruda et al., 2011; Tran et al., 2016).

Insulin promotes its anorexigenic signal in the hypothalamus by Forkhead Box O1 (FoxO1) transcription factor phosphorylation through the phosphatidylinositol 3-kinase (PI3K)/protein kinase B (Akt) pathway (Ropelle et al., 2010; Rodrigues et al., 2015). When dephosphorylated, FoxO1 is colocalized in the cell nucleus acting on the orexigenic neuropeptides genes transcription, such as Neuropeptide Y (NPY) and Agouti-related peptide (AgRP), resulting in hyperphagia (Ropelle et al., 2010; Rodrigues et al., 2015). Mitogen-activated Protein Kinase Phosphatase 3 (MKP-3) has been described as one of the main FoxO1 activators due to its affinity to dephosphorylate this protein (Xu et al., 2005; Wu et al., 2010; Feng et al., 2012; Jiao et al., 2012; Pauli et al., 2014). Previously, it was described in the literature that the MKP-3 content is increased in the hepatic tissue of obese rodents, which results in FoxO1 dephosphorylation and hepatic glucose production increase (Xu et al., 2005; Wu et al., 2010; Jiao et al., 2012; Pauli et al., 2014). Also, the overexpression of MKP-3 in the hypothalamus of lean mice decreased FoxO1 phosphorylation, which suggests the participation of this phosphatase on food intake (Rodrigues et al., 2017).

Also, insulin activates the Mitogen-activated protein kinase kinase (MEK), and Extracellular signal-regulated kinase (Erk) pathway, which contributes to the NPY/AgRP transcription inhibition (Mayer and Belsham, 2009). The hypothalamic phosphorylation of Erk has great importance on energy metabolism, once it is necessary for the thermogenesis regulation (Rahmouni et al., 2009). It is well known that MKP-3 is one of the main Erk inactivators (by dephosphorylation; Feng et al., 2012, 2014a,b; Ndong et al., 2014). However, these mechanisms of interaction among MKP-3, FoxO1 and Erk have not been investigated in the central nervous system level, especially in the hypothalamus in obesity condition. In the present study, for the first time, we investigated the effects of high-fat diet-induced obesity on MKP-3 content, and FoxO1 and Erk phosphorylation in the hypothalamus of mice. Also, the food intake behavior and energy expenditure were measured in these obese mice.

## Materials and Methods

### Experimental Animals

Swiss mice (6 weeks old) were distributed in individual cages with free access to water and food. The animals were divided between the lean group (Lean) that received standard commercial diet (composed of 5 g of fat, 22 g of protein and 51.5 g of carbohydrate in 100 g of diet–339 of total kcal) from Presence^®^, and the obese group (Obese) that received high-fat diet (composed of 35.8 g of fat, 23 g of protein and 34.5 g of carbohydrate in 100 g of diet—552.2 of total kcal; Cintra et al., 2012). Six to 10 animals were used per experimental group, and all of them were conditioned in a controlled environment with temperature (21°C ± 2) and photoperiod (12/12 h light/dark). The Animal Use Ethics Committee (CEUA) of State University of Campinas—UNICAMP (No. 4064-1), approved the experimental procedures.

### Insulin Tolerance Test (ITT) and Glucose Tolerance Test (GTT)

At the end of the experimental period, the physiological tests were performed with animals after the 8-h fasting period. For Insulin Tolerance Test (ITT) test, a dose of 2 U/Kg insulin (intraperitoneal) was applied to the first blood collection, and new samples were collected from the animal’s tail at 5, 10, 15, 20, 25 and 30 min for the blood glucose determination. The constant rate of glucose decrease (Kitt) was calculated (Microsoft Excel 2013^®^) using the formula 0.693/t1/2 and through the trapezoidal method as previously described (Botezelli et al., 2016). For Glucose Tolerance Test (GTT), after blood collection to determine the initial blood glucose, a dose of 2 g/Kg glucose (intraperitoneal) was applied, and new samples were collected from the animal’s tail after 30, 60 and 120 min. The area under the curve (AUC) was calculated by glucose values mean through the trapezoidal method (Microsoft Excel 2013^®^; Botezelli et al., 2016). Also, fasting blood glucose values were obtained through the first ITT collection point.

### Hypothalamus Extraction and Immunoblotting Analysis

The animals received anesthesia (ketamine/200 μL/Kg and xylazine/400 μL/Kg) and diazepam (200 μL/Kg)) intraperitoneally. Then, insulin (200 mU) was administrated via i.c.v. by microinjection (stereotactic coordinates: anteroposterior −1.8 and depth −5.0) 5 min before the euthanasia. The procedures for the hypothalamus extraction and immunoblotting analysis have been previously described by our research group (Silva et al., 2014). The primary antibodies used were anti-Akt (rabbit, sc-8312, Santa Cruz Biotechnology^®^), anti-phospho-Akt (Ser473; rabbit, sc-101629, Santa Cruz Biotechnology^®^), anti-FoxO1 (rabbit, 9454s, Cell Signaling Technology^®^), anti- β-Actin (rabbit, 8457, Cell Signaling Technology^®^), Anti-phospho-Erk1/Erk2 (T202/Y204/T185/Y187; rabbit, AF1018, R&D Systems^®^), anti-Erk1/2 (rabbit, AF1576, R&D Systems^®^), anti-phospho-FoxO1 (Ser256; rabbit, 9461s, Cell Signaling Technology^®^), anti-MKP-3 (rabbit, bs-11546R, Bioss^®^). The luminescence of the samples present in the membranes was detected by a chemiluminescent reagent (ECL) and subsequently exposed to RX (Kodak^®^) films and quantified by densitometry (UN-SCAN-IT gel 6.1 software).

### Energy Expenditure and Food Intake Analysis

The Comprehensive Lab Animal Monitoring System (CLAMS)–Columbus Instruments^®^ was used for the analysis of oxygen consumption (VO_2_), heat production, spontaneous activity and respiratory exchange rate (RER). The animals were adapted during 24 h to the equipment, and in the following 24 h, the data were measured for later analysis. For the food intake analysis, the animals received insulin stimulation (200 mU) via i.c.v. microinjection and the food amount consumed during the 12 h period was recorded (3.17).

### Bioinformatics Analysis

Bioinformatics analysis was performed using a dataset from hypothalamus of genetically-diverse BXD male mice (INIA Hypothalamus Affy MoGene 1.0 ST (Nov10) Male; Mozhui et al., 2012) and VO_2_ (maximal oxygen consumption) during the light phase and body weight values were obtained using a dataset as previously published (Williams et al., 2001, 2016). These data sets are accessible on Genetwork^1^ from the largest dataset and categorized studies involving transcriptome and phenotype analysis from BXD mice strains (Andreux et al., 2012). The Pearson’s correlation graphs were elaborated using GraphPad Prism 6.01^®^. Complete data used for analysis is on S1.

### Statistical Analysis

The data are presented as means and standard errors of the mean (SEM), and the normality distribution was accessed using the Shapiro-Wilk W-test. When the data were normal, Student’s *t*-test was performed. For non-parametric data, the Mann-Whitney test was used. About the line graphs, the *two-way* analysis of variance (ANOVA) test was performed with repetitive analysis to evaluate the points separately. The values of *p* < 0.05 were considered statistically significant, *p* < 0.01 were considered very significant and *p* < 0.001 represented extremely significant power. The statistical tests were performed using GraphPad Prism 6.01^®^.

## Results

### Metabolic and Physiological Parameters

**Figure 1:**
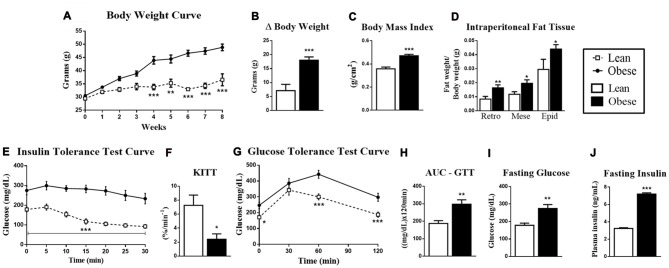
Metabolic and physiological parameters of lean and obese animals. **(A)** Body mass curve during 8 weeks. **(B)** Δ Body mass between the first and eighth week of treatment. **(C)** Body mass index (BMI; g/cm^2^). **(D)** The retroperitoneal, mesenteric and epididymal fat mass weight/body weight. **(E)** Insulin Tolerance Test (ITT) curve. **(F)** ITT KITT curve. **(G)** Glucose Tolerance Test (GTT) curve. **(H)** Area under the curve (AUC) of the ITT. **(I)** Fasting glucose. **(J)** Fasting insulin. The bars and lines in the graphs represent the mean and standard error of the mean (SEM; *n* = 6–10). *p < 0.05 vs. Lean group. **p < 0.01 vs. Lean group. ***p < 0.001 vs. Lean group.

The first step of our study was to characterize the obese group in relation to the healthy group. Thus, we observed that obese animals showed an increased body mass from the fourth week of obesity induction, with changes in final body mass, body mass index (BMI) and weight variations between the first week and the eighth week of treatment (Δ body weight; Figures 1A–C). In the same way, obese animals had fat accumulation on intraperitoneal, retroperitoneal, mesenteric and epididymal tissues (Figure 1D). These morphological changes resulted in the insulin resistance aggravation, which was observed by the ITT (Figures 1E,F), and glucose intolerance, which was observed in the AUC for the GTT (Figures 1G,H). Also, these animals presented hyperglycemia and fasting hyperinsulinemia (Figures 1I,J).

### Obese Animals Had an Increased Hypothalamic MKP-3 Content

**Figure 2:**
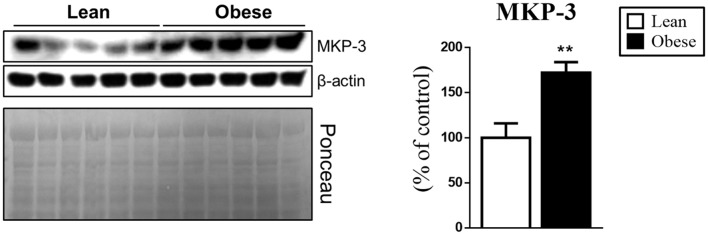
Increased hypothalamic Mitogen-activated Protein Kinase Phosphatase 3 (MKP-3) content in obese mice. MKP-3 protein content in the hypothalamus of lean and obese mice. The bars in the graphs represent the mean and SEM (*n* = 5). **p < 0.01 vs. Lean group.

After the characterization of the obese group, the next step was to evaluate if the obesity changes were associated with the MKP-3 increase in the hypothalamic tissue. For this, we evaluated the hypothalamus of the different groups, where the obese group showed a significant increase of MKP-3 (by 72%, *t* = 3.635, *df* = 8, *p* = 0.0066) compared to the lean animals (Figure 2).

### The Increased Hypothalamic MKP-3 Content Is Associated with FoxO1 and Erk1/2 Phosphorylation

**Figure 3:**
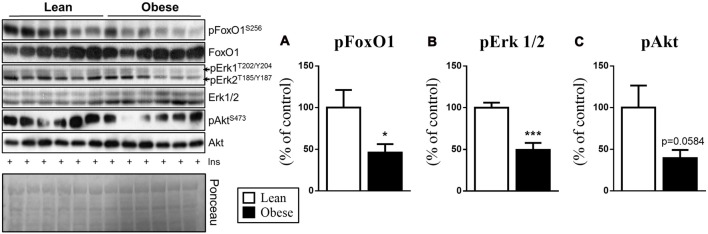
Phosphorylation of the proteins involved with the MKP-3 phosphatase activity (pFoxO1 and pErk1/2) after insulin stimulus. **(A)** FoxO1 phosphorylation (S256) normalized by its total content. **(B)** Erk1/2 phosphorylation (T202/Y204/T185/Y187) normalized by its total content. **(C)** Akt phosphorylation (S473) normalized by its total content. Related to the total content of FoxO1, Erk and Akt were used as a control, only Erk1/2 showed a significant increase compared to the CTL group. The bars in the graphs represent the mean and SEM (*n* = 6). *p < 0.05 vs. Lean group. ***p < 0.001 vs. Lean group.

The importance of the MKP-3 phosphatase activity on FoxO1 and Erk1 2 is well described; therefore, we evaluated the phosphorylation of these proteins in the obesity condition. Thus, in obesity, the animals presenting an increase in the hypothalamic MKP-3 also demonstrated a decrease in the phosphorylation of FoxO1 (by 54%, *t* = 2.88, *df* = 10, *p* = 0.0451) and Erk1/2 (by 51%, *t* = 4.900, *df* = 8, *p* = 0.0006; Figures 3A,B), corroborating our previous data. Also, the obese group showed a strong tendency of decreased Akt phosphorylation (by 61%, *t* = 2.137, *df* = 10, *p* = 0.0584) in relation to the control group, which demonstrates the insulin signaling pathway impairment in response to obesity induction.

### Heat Production and Food Intake Are Impaired in Obese Mice

**Figure 4:**
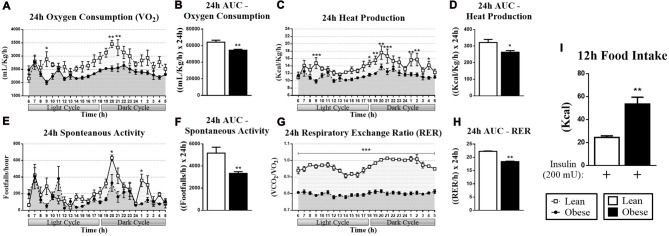
Respirometry and dietary intake data of lean and obese mice. **(A)** Oxygen consumption (VO_2_) during 24 h. **(B)** AUC for the VO_2_ during 24 h. **(C)** Heat production measured during 24 h. **(D)** AUC for the heat production during 24 h. **(E)** Spontaneous activity during 24 h. **(F)** AUC for the spontaneous activity during 24 h. **(G)** Respiratory exchange ratio (RER) during 24 h. **(H)** AUC for the RER during 24 h. **(I)** Cumulative food intake of 12 h after insulin stimulation. The bars and lines in the graphs represent the mean and SEM (*n* = 6). *p < 0.05 vs. Lean group. **p < 0.01 vs. Lean group. ***p < 0.001 vs. Lean group.

The increase in the hypothalamic MKP-3 content is associated with decreased phosphorylation of two important proteins of the energy control (pFoxO1 and pErk1/2), probably due to its phosphatase activity. Since the hypothalamic Erk1/2 phosphorylation is directly related to energy control, we submitted the lean and obese animals to the respirometry analysis during 24 h. Thus, in comparison with the healthy rodents, we observed that obese animals showed lower levels of oxygen consumption (Figures 4A,B), heat production (Figures 4C,D), spontaneous activity (Figures 4E,F) and RER (Figures 4G,H).

Regarding FoxO1 phosphorylation, its decrease is associated with an increase in food intake, but these data had to be confirmed. Then, the dietary intake of lean and obese mice was measured by injecting 200 mU of insulin directly into the arcuate nucleus for 12 h. Figure 4 shows that the obese animals increased the food intake compared to the lean group.

### Bioinformatics Analysis Reinforces that MKP-3 Is Related to Energetic Homeostasis Maintenance

**Figure 5:**
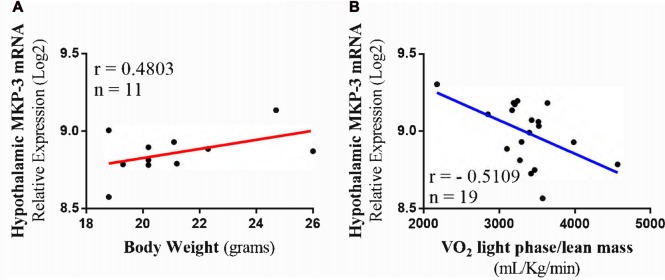
Transcriptome and phenotype bioinformatics analysis. **(A)** Positive Pearson’s correlation between hypothalamic MKP-3 mRNA in lean mice and body weight (*n* = 11). **(B)** Negative Pearson’s correlation between hypothalamic MKP-3 mRNA and oxygen consumption (VO_2_) in light phase (*n* = 19).

To extend our findings of the correlations between MKP-3, oxygen consumption and body weight, we performed transcriptome and phenotype analysis using a database with hypothalamic samples of BXD strains. For this, we used a transcriptome dataset from the largest and best-categorized study involving genetically-diverse BXD mice strains (Andreux et al., 2012). The analysis using lean mice phenotype strains (*n* = 11) showed that MKP-3 mRNA level was positively correlated with the body weight (*r* = 0.4803; Figure 5A). On the other hand, those strains with higher MKP-3 mRNA levels (*n* = 19) presented a negative correlation with oxygen consumption during light phase corrected by the lean mass (−0.5109; Figure 5B). These results suggest that MKP-3 is an important molecule for the energetic homeostasis maintenance. Taken together, we suggest that the increased MKP-3 levels can result on positive, energetic balance.

## Discussion

The hypothalamic insulin signaling plays an important effect on the food intake control. Phosphorylation and inactivation of FoxO1 protein is a fundamental step in this pathway, once this protein is responsible for the orexigenic neuropeptides transcription, such as NPY and AgRP. On the other hand, MKP-3 can dephosphorylate and activate FoxO1, which allows its translocation to the nucleus initiating its transcriptional activity. However, this mechanism had not been demonstrated in the hypothalamic tissue of obese rodents. Several results in the literature suggest that obesity is closely related to increased MKP-3 content in peripheral tissues in both transgenic models (ob/ob; Wu et al., 2010) as well as through high-fat diet induction (Wu et al., 2010; Pauli et al., 2014). In the same way, it is a consensus that FoxO1 phosphorylation is reduced in this pathological condition and MKP-3 may play a key role in this phenomenon.

One of the main proteins responsible for the phosphorylation and inactivation of FoxO1 after insulin stimulation is Akt (Ropelle et al., 2010; Rodrigues et al., 2015). It is well known that one of the major hypothalamic molecular disorders attributed to obesity is the insulin inability to propagate its intracellular signal, which results in lower Akt phosphorylation (Ropelle et al., 2010; Rodrigues et al., 2015). Our results reinforce the previous statement (Figure 3C). After the FoxO1 phosphorylation by Akt, this transcription factor is extruded from the nucleus to the cytoplasm, being marked for further degradation by ubiquitination (Aoki et al., 2004; Coppari et al., 2009).

Another substrate for MKP-3 action is Erk (Ndong et al., 2014), a protein necessary for the insulin biological effect in the transcription inhibition of orexigenic neuropeptides (Mayer and Belsham, 2009). Administration of Erk’s pharmacological inhibitor in hypothalamic cells (mHypoE-46) impaired insulin ability to reduce NPY and AgRP mRNA levels (Mayer and Belsham, 2009). Transcriptional factors binding sites analysis revealed that after phosphorylation by insulin stimulation, Erk activates transcription factors that can bind regulatory regions of NPY and AgRP 5’, suppressing the gene transcription of these orexigenic neuropeptides (Mayer and Belsham, 2009). Hypothalamic Erk phosphorylation is also related to increased thermogenesis. Also, after its pharmacological inhibition, there was a reduction in thermogenesis and brown adipose tissue activation by the hypothalamus (Rahmouni et al., 2009).

Therefore, considering important roles of FoxO1 and Erk in the control of food intake, we evaluated the phosphorylation of these two proteins in response to insulin stimulation in lean and obese animals. Obese animals demonstrated an increased MKP-3 content in the hypothalamus as well as a decreased FoxO1 and Erk1/2 phosphorylation (Figures 3A,B). These data corroborate our previous study in which MKP-3 overexpression led to FoxO1 dephosphorylation, suggesting a relationship between MKP-3 phosphatase activity and these targets in the hypothalamic tissue during the obesity condition (Rodrigues et al., 2017). Also, the reduced phosphorylation of hypothalamic FoxO1 and Erk1/2 impaired the energy metabolism and elevated the food intake (Mayer and Belsham, 2009; Rahmouni et al., 2009). Then, we investigated whether these molecular mechanisms were linked to metabolic and physiological changes in the obesity condition. We observed that obese mice presented lower oxygen consumption, heat production, spontaneous activity and RER during a 24-h period (Figures 4A–H). Regarding the RER, we observed that the lean group presented values close to 1, which suggests that mainly carbohydrates were used as an energy substrate (Lam and Ravussin, 2017). On the other hand, the obese animals presented values close to 0.8, suggests that mainly lipids were used as an energy substrate (Lam and Ravussin, 2017). Finally, the food intake of the obese animals was higher during 12 h after the insulin stimulation (Figure 4I). Such disturbances were accompanied by increased levels of body weight and adiposity, decreased levels of insulin sensitivity, and glucose intolerance.

Finally, our transcriptome analysis reveals that the MKP-3 mRNA expression was associated with a positive energetic balance, suggesting the role of this protein in body weight gain and energy expenditure reduction. The involvement of MKP-3 levels in energy homeostasis is associated with several metabolic derangements by different mechanisms (Xu et al., 2005; Wu et al., 2010; Jiao et al., 2012; Feng et al., 2014a). However, regarding humans studies, there is no evidence involving the hypothalamic tissue and MKP-3 content. Then, cell culture and animal studies try to elucidate the role of this protein as a promising molecule with therapeutic action against obesity. Other studies are necessary to investigate possible interventions capable of attenuating the MKP-3 action in the human hypothalamus.

**Figure 6:**
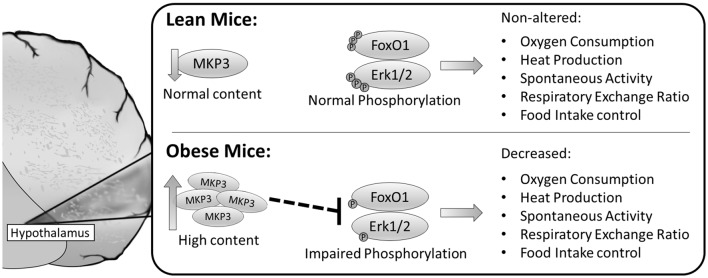
Schematic model summarizing the possible molecular mechanisms by which MKP-3 led to food intake increase and thermogenesis decrease. The obese mice presented high content of MKP-3 in the hypothalamus, and decreased phosphorylation of FoxO1 and Erk1/2 leading to decreased oxygen consumption, heat production, spontaneous activity, RER and increased food intake.

In summary, our findings contribute to new evidence on the hypothalamic molecular mechanisms involved in the imbalance of energy homeostasis and food intake in obesity and insulin resistance. The potential molecular mechanisms by which MKP-3 led to food intake increase and thermogenesis decrease are summarized in Figure 6. Therefore, taking our results together, the MKP-3 increased in the hypothalamus, at least in part, contributing to the insulin signaling pathway impairment due to its effect on FoxO1 and Erk dephosphorylation, which induce hyperphagia and obesity in animals.

## Author Contributions

BAR, GKK and RCG were responsible for the experimental procedures and data acquisition. VRM and GKK were responsible for the manuscript writing, the data analysis and the design of the figures. BMC was responsible for the transcriptome analysis. SCBRN was responsible for the manuscript writing. JDB, LSSP and ASRS were responsible for the manuscript review. LPM, DEC and ERR were responsible for the manuscript review and the laboratory support. JRP was responsible for the laboratory support, the research funding, the manuscript design and the final manuscript review.

## Conflict of Interest Statement

The authors declare that the research was conducted in the absence of any commercial or financial relationships that could be construed as a potential conflict of interest.

## Footnotes

^1^http://www.genenetwork.org
